# January

**Published:** 1890-01-04

**Authors:** 


### JANUARY.

Merriment without discretion is an abuse for which Nature is eventually sure to punish us. Therefore let us hope we have all exercised discretion during the past Christmas and New Year festivities, so that we may commence the year 1890 with the *mens sana in corpore sano*. But there is still something to do, in order that we may secure this desirable condition of body and mind throughout the twelvemonth just commenced. Although the sun may now begin to gain power, and the days to lengthen, the maximum of cold generally follows the shortest day, for, as the old adage has it, "when the days begin to lengthen the cold begins to strengthen." Astronomers tell us that at the commencement of January the earth is at its least distance from the sun. *A priori*, it would, therefore, appear that this should be the warmest period of the year. But the coldness of winter does not at all depend upon the mere distance of the earth from the sun. It depends on relative position, and on the very oblique and slanting direction of the rays of the sun at this season. There is also another potent cause of cold in the shortness of the days, and the length of the nights, the sun being below our horizon at the beginning of January about sixteen hours and a half daily. There is still another cause in the prevalence of north-east winds, with which come storms and snow. It is common observation that easterly winds are detrimental. Why this should be the case we have never seen a satisfactory explanation. It behoves us, however, to accept the fact, and to guard against it. Especially so, when we draw near that period of life when waist girth begins to exceed chest girth. Or when, as an old author described the signs of age, "we begin to laud the past and decry the present, to read with glasses, to recoil from change, to find sense in twaddle, to know the value of health from the fear of losing it, to feel an interest in rheumatism, an awe of bronchitis, to tell anecdotes, and to wear flannel." To be young, to be hungry, to be able to walk five miles an hour, are advantages of which men are scarcely conscious while they possess them. And to this may be added the capability of defying the cold and east winds of January. It is said there are sermons in stones and good in everything, even in January east winds. For they at least drive the fogs away, from which we so generally suffer during the two preceding months. Some may think the latter are the least of the two evils, and especially some asthmatics, who are never better than when inhaling a London fog. Leigh Hunt described a January morning thus: "On opening my eyes the first thing that meets them is my own breath rolling out like smoke from a cottage chimney. Then I turn my eyes sideways and see the window all frozen over." So he stays in bed for a time, meditating on the "unnecessary and villainous custom of shaving," which, by the bye, was more habitual with our forefathers than it is now. But remarkable instances of the mildness of January have been recorded. Some years back a couple of mistaken blackbirds built their nest and hatched four eggs, strawberries bloomed, and the fruit ripened, the usual spring flowers appeared, and bees flew about as in the summer, while robins disdained to come as supplicants to the window.

The zodiacal sign of January is Aquarius, or the water bearer, emblematic of the rain we may expect. It derives its name from Janus, a deity represented by the Romans with two faces, because he was acquainted with past and future events, and January may be regarded as the link between the past and the future years. The "skirts of the departing year" vanish as the new year is born. New Year's Day was formerly a period of great merriment, emphasised by the giving of gifts. In these degenerate days we do not observe it as any great public festival, although the custom of giving presents has not entirely died away. Most of us, however, are rather sad than glad on New Year's Day, for we cannot help thinking that another year is passed from our allotted span of life, and whatever may be said to the contrary, the great majority do think that life is worth living. January 5, or Twelfth Night Eve, was also formerly a notable day. It was once the practice in country places to dance round the apple trees, asking them to bear "pocket-fulls, hat-fulls, peck-fulls, bushel-bag-full." And on January 6th people were wont to make themselves ill by eating Twelfth cake. Now, however, Christmas festivities must end and sober business resume its sway. Thus the first Monday after Twelfth Day was called "Plough Monday," because it was the first day after Christmas that cultivators resumed the plough. In some parts of the country the plough, decorated with such flowers as could be procured, was dragged from door to door, of course with the idea of obtaining vails to spend in beer. January is rich in saint days, but, says an old chronicle, "of the seven Romish saints of this day, January 9th, there is scarcely an anecdote worth mentioning." January 17th was St. Anthony's Day. This saint passed through many temptations, and enjoyed the reputation of curing inflammations. January 25 is St. Paul's Day, when formerly prognostications for the whole year were drawn, and we have the old couplet,

"If St. Paul's Day be fair and clear
It does betide a happy year."

No one, however, seems to know why this day in particular should have been held favourable for prognostication and fortune telling, and Gay said, "Let no such vulgar tales debase thy mind, nor Paul nor Swithin rule the clouds and wind." In former days, weather prophets were numerous, and many seem to have fixed on a certain day from the appearance of which they professed to foretell the weather. In these times most people pin their faith on the weather forecasts as published in the *Times*, and it must be allowed that these forecasts are sometimes correct.

But even so early as the end of January there are indications of returning life and spring. The attentive observer may find embryo buds in their silky, downy coats, finely varnished, to protect them from the wet and cold. As Cowper wrote, "from death to plenty and from death to life is nature's progress" at this season of the year. It will be some time before the embryo buds appear fresh and green, but they are there, and "mark the bounds which winter may not pass." They may be delayed by frost, and cold, and snow, but eventually they will achieve the victory, for they are well taken care of by their nurse Nature. As human beings are not exactly winter flowers like the snowdrop or the crocus, it behoves them also to follow the example of Dame Nature in her care for her buds, and to protect themselves also. To appreciate the vernal signs we should endeavour to remove ourselves back to the earlier condition of our race, when warm clothing was a luxury of the great; when the mass of the people lived in mere huts; when, in comparison with the present day, all were badly fed. Winter was then a ruthless tyrant indeed. We can scarcely be surprised at the prominence given by the nations of old to rejoicings, even so early as the birth of the new year, on the prospect of returning sun-power. We have not yet succeeded in averting all the destructive influences of cold and darkness, although a good deal has been effected. It is at this season of the year that the claims of the poor press heavily on the public.

